# TRP Channel Agonists Activate Different Afferent Neuromodulatory Mechanisms in Guinea Pig Urinary Bladder

**DOI:** 10.3389/fphys.2021.692719

**Published:** 2021-06-24

**Authors:** Stephanie L. Daugherty, Jonathan M. Beckel, Kyoungeun A. Kim, Bruce A. Freeman, Jiaxin Liu, Shaoyong Wang, William C. de Groat, Xiulin Zhang

**Affiliations:** ^1^Department of Pharmacology and Chemical Biology, University of Pittsburgh School of Medicine, Pittsburgh, PA, United States; ^2^Department of Urology, The Second Hospital, Cheeloo College of Medicine, Shandong University, Jinan, China

**Keywords:** nitro-oleic acid, afferent nerves, TRP channels, guinea pig, urinary bladder

## Abstract

Activation of TRP channels expressed in urinary bladder afferent nerves and urothelium releases neurotransmitters that influence bladder function. Experiments were undertaken to examine the mechanisms underlying effects of TRPA1 (allyl isothiocyanate, AITC), TRPV1 (capsaicin, CAPS), and TRPC (oleoyl-2-acetyl-sn-glycerol, OAG) agonists on guinea pig bladder activity. Effects of these agonists were compared with effects of nitro-oleic acid (OA-NO_2_), an electrophilic nitro-fatty acid, known to activate TRPV1, TRPA1 or TRPC channels in sensory neurons. AITC (100 μM) increased (231%) area of spontaneous bladder contractions (SBCs) an effect reduced by a TRPA1 antagonist (HC3-03001, HC3, 10 μM) and reversed to inhibition by indomethacin (INDO, 500 nM) a cyclooxygenase inhibitor. The post-INDO inhibitory effect of AITC was mimicked (39% depression) by calcitonin gene-related peptide (CGRP, 100 nM) and blocked by a CGRP antagonist (BIBN, 25 μM). CAPS (1 μM) suppressed SBCs by 30% in 81% of strips, an effect blocked by a TRPV1 antagonist (diarylpiperazine, 1 μM) or BIBN. SBCs were suppressed by OA-NO_2_ (30 μM, 21% in 77% of strips) or by OAG (50 μM, 30%) an effect blocked by BIBN. OA-NO_2_ effects were not altered by HC3 or diarylpiperazine. OA-NO_2_ also induced excitation in 23% of bladder strips. These observations raise the possibility that guinea pig bladder is innervated by at least two types of afferent nerves: [1] Type A express TRPA1 receptors that induce the release of prostaglandins and excite the detrusor, [2] Type B express TRPV1, TRPA1 and TRPC receptors and release CGRP that inhibits the detrusor.

## Introduction

The urinary bladder is innervated by small myelinated (Aδ) and unmyelinated (C-fiber) afferent nerves that respond to mechanical and chemical stimuli and then transmit information to the central nervous system about bladder filling and the chemical environment in the bladder (de Groat et al., 2015). Activation of C-fiber bladder afferents also releases neurotransmitters that act on the smooth muscle to directly regulate bladder contractility (Maggi, 1993). This “efferent function” of bladder afferent nerves is mediated in part by neuropeptides (Maggi and Meli, 1986; Maggi, 1991, 1993). Thus, bladder afferents are classified, based on receptors and transmitters that they express as well as by axonal size and myelination.

A common type of bladder afferent has an unmyelinated axon, expresses multiple types of transient receptor potential (TRP) channels and synthesizes and releases neuropeptides such as neurokinins or calcitonin gene-related peptide (CGRP) (de Groat and Yoshimura, 2009). In many species these afferents respond to agonists for TRPV1 (capsaicin, CAPS) (Maggi and Meli, 1986; de Groat and Yoshimura, 2009) or TRPA1 receptors (allyl isothiocyanate, AITC) by releasing neurotransmitters that enhance (Maggi, 1993; Andrade et al., 2006; Artim et al., 2011) or inhibit contractions (Maggi, 1993; Gillespie, 2005) of the bladder smooth muscle. In rats the excitatory effect is also mimicked by application of OA-NO_2_, a thiol-reactive electrophilic nitro-fatty acid (Artim et al., 2011) that mediates the post-translational modification of susceptible protein thiols, thus activating TRPV1 and TRPA1 receptors in rat dorsal root ganglion (DRG) cells (Sculptoreanu et al., 2010; Zhang et al., 2014b). OA-NO2 or other nitro-fatty acids which have exhibited anti-inflammatory properties in both animal and clinical studies (Rudolph et al., 2010; Freeman et al., 2018; Schopfer et al., 2018) may find use in the future in the treatment of inflammatory or painful bladder conditions.

However, the effect of OA-NO_2_ on sensory nerves is complicated because this thiol-reactive electrophile will target different receptors in different afferent nerves and in different species. While OA-NO_2_ activates TRPV1 and TRPA1 both in rat DRG neurons (Sculptoreanu et al., 2010) and bladder afferent nerves (Artim et al., 2011), OA-NO_2_ selectively targets TRPA1 in mouse vagal afferent neurons (Taylor-Clark et al., 2009) and targets TRPV1, TRPA1, or TRPC in guinea pig DRG neurons (Zhang et al., 2014a).

The present experiments were undertaken to determine the mechanisms underlying effects of OA-NO_2_ on the guinea pig bladder and to compare its effects with those of more selective activators of TRPA1, TRPV1, and TRPC (oleoyl-2-acetyl-sn-glycerol, OAG). The effects of four agonists (CAPS, AITC, OAG, and OA-NO_2_) were examined on spontaneous activity of bladder strips with intact mucosa from adult guinea pigs.

## Materials and Methods

### Bladder Strip Preparation

All experimental procedures were approved by the Institutional Animal Care and Use Committee of the University of Pittsburgh. Bladder strips from adult 200 to 400g male (*n* = 11) and female (*n* = 47) Hartley guinea pigs (Charles River Wilmington, MA) were prepared as described previously (Artim et al., 2011; Kullmann et al., 2014). Under isoflurane anesthesia (4% in O_2_) the bladders were removed and placed in room temperature Krebs solution (composition in mM: NaCl 118, KCl 4.7, CaCl_2_ 1.9, MgSO_4_ 1.2, NaHCO_3_ 24.9, KH_2_PO_4_ 1.2, dextrose 11.7; pH. 7.4, bubbled with 95% O_2_ and 5% CO_2_). Each bladder with the mucosa intact was cut into eight longitudinal strips, extending from the bladder neck to the dome and were mounted in a vertical double-jacketed organ bath with oxygenated Krebs solution (15-ml volume) and kept at 37°C via a circulating water pump. Tissue was stretched by applying 10 mN (1 g) baseline tension and allowed to equilibrate for 1–2 h prior to drug testing. Antagonists were applied in the bath 15–60 min prior to or following agonist application.

### Chemicals

Chemicals used in this study include: nitro-oleic acid [OA-NO_2_, a mixture of the two possible regioisomers of the nitroalkene substituent (E)-9- and 10-nitro-octadec-9-enoic acid] synthesized as described previously (Baker et al., 2005); oleic acid (30 μM) a non-nitrated fatty acid; capsaicin (CAPS, 1 μM), a TRPV1 agonist; allyl isothiocyanate (AITC, 100 μM), a TRPA1 agonist; diarylpiperazine (DP, 1 μM), a selective TRPV1 antagonist (Ki = 6 nM for inhibition of acid pH evoked responses and 35 nM for inhibition of CAPS evoked responses) (Sculptoreanu et al., 2010; Artim et al., 2011; Zhang et al., 2014a), a gift from Neurogen Corp (Branford, CT, USA); HC3-03001 (HC3, 10 μM), a selective TRPA1 antagonist (a gift from Hydra Biosciences, Inc., Cambridge, MA); and three neurokinin antagonists (subtypes 1, 2, and 3: SR 140333, SR 48968, and SR 142801, respectively, 1 μM each) (Holzer et al., 1998; Takahashi et al., 2002; Wise et al., 2007), gifts from Sanofi-Aventis LLC (Bridgewater, NJ), were applied in combination. All other agents: tetrodotoxin (TTX, 1 μM), lanthanum (III) chloride heptahydrate (La^3+^, 50 μM), 1-oleoyl-2-acetyl-sn-glycerol (OAG, 50 μM), human α calcitonin gene-related peptide (CGRP, 100 nM), olcegepant (BIBN, 25 μM), substance P (SP, 1 μM), prostaglandin E_2_ (500 nM) and indomethacin (INDO, 500 nM) were obtained from Sigma Aldrich (St. Louis, MO) or Tocris (Bristol, UK). Vehicles: distilled water for CGRP and SP; DMSO (0.05% final concentration) for all other substances, did not significantly change the activity of bladder strips. Chemicals were used in concentrations shown to be effective in preliminary experiments or in concentrations used in previously published studies (Maggi et al., 1988a,b; Gillespie, 2005; Artim et al., 2011; Zhang et al., 2014a).

### Data Analysis

All data are presented as mean ± S.E.M. To study the onset and duration of agonist-evoked responses, majority of the bladder strip activity was measured in 5 min intervals immediately before (basal activity) and at varying intervals after application of the agonists, during the time period when they exerted their maximal or prolonged effects: [a] 0–30 s for CAPS early transient response (compared to a 30 s interval immediately before application of CAPS), [b] 0-5 min for OA-NO_2_, CAPS, SP, and CGRP, [c] 10–15 min for AITC and prostaglandin E_2_, [d] 0–65 min for OA-NO_2_, AITC, CAPS, and OAG. The effects were measured as a change in area under the curve (AUC) which reflects changes in amplitude and frequency of SBCs and as a change in baseline tension. Data are reported as percent change from basal activity. Eighty percent of the experiments were conducted on tissue from female animals; and the data from both sexes were combined for data analysis because strips from male and female guinea pig did not exhibit obvious differences in response to agonists and antagonists. Statistical significance was tested on raw data using a paired two tailed *t*-test, ^*^P < 0.05 and ^**^P < 0.001, a one-way ANOVA with Dunnett's multiple comparison *post-hoc* test (^+^*P* < 0.05) or a two-way ANOVA and Bonferroni multiple comparisons *post-hoc* test (brackets, ^#^*P* < 0.05) when appropriate. Excel and Graph Pad Prism 7 software were used for analysis. Data were considered statistically significant when *P* < 0.05.

## Results

### AITC, a TRPA1 Agonist, Enhances SBCs

**Figure 1:**
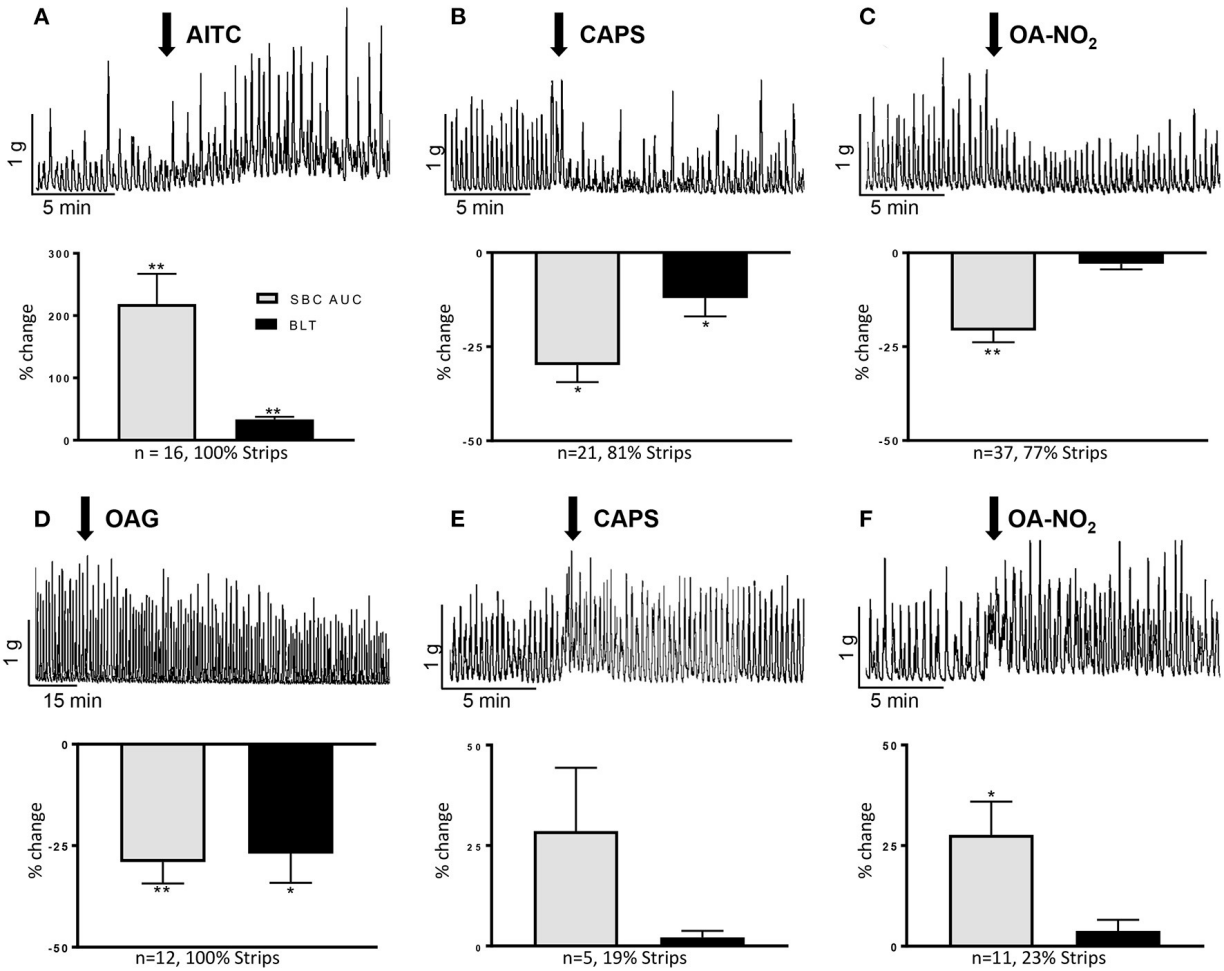
Effects of AITC (100 μM), capsaicin (CAPS, 1 μM), OA-NO_2_ (30 μM), and OAG (50 μM) on guinea pig spontaneous bladder contractions (SBCs). **(A–C)** Upper traces show the common excitatory response of AITC and inhibitory responses of CAPS and OA-NO_2_. Drugs were applied at the arrows and maintained in the tissue bath. Graphs summarize the major responses to the three agonists as % change in SBC area under the curve (AUC) compared to basal activity, baseline tension (BLT) and the percentage of strips exhibiting the responses. **(D–F)** Upper traces show the slow onset suppression of SBCs by OAG and the less common excitatory effects of CAPS and OA-NO_2_. Graphs summarize the responses of the three agonists as % change in SBC area under the curve (AUC) compared to basal activity, baseline tension (BLT) and the percentage of strips exhibiting the responses. Calibration bars refer to time (min) and tension (gram, g). Statistics were performed on raw numbers: 2-tailed *t*-test, *P < 0.05 and **P < 0.00, compared with basal activity; *n*, number of strips.

When stretched with 1 g tension, guinea pig bladder strips displayed spontaneous bladder contractions (SBCs) of variable amplitude and frequency (Figures 1A–F) that persisted over many hours. Consistent with its excitatory effect in rat bladder strips (Andrade et al., 2006; Artim et al., 2011) the TRPA1 agonist AITC (100 μM) increased AUC of SBCs by 231 ± 47% and baseline tension by 37 ± 6% in all strips (*n* = 16 strips from nine female and two male guinea pigs; *P* <0.001; Figure 1A). The excitatory effect reached a maximum at 4-5 min after application and persisted for over 30 min.

### CAPS, a TRPV1 Agonist, Has Bimodal Facilitatory and Inhibitory Effects on SBCs

CAPS at a concentration (1 μM) that enhances rat SBCs (Artim et al., 2011), elicited a bimodal response consisting of a transient excitation lasting on average 35 s that increased the AUC of a few contractions by 128 ± 50% (*P* < 0.002) followed by a more prominent inhibition in 81% of 26 strips from 1 male and 16 female guinea pigs (Figure 1B). The inhibition decreased the AUC by 30 ± 5% and baseline tension by 11 ± 4% (*P* < 0.01, Figure 1B). Inhibition reached a maximum at an average of 2 min after application and persisted for 25–30 min. In the remaining strips CAPS increased the SBCs by 29 ± 16% for a period of several minutes (Figure 1E). However, the magnitude of the prolonged excitatory effect was variable between experiments and the average increase in the AUC was not statistically significant (*P* > 0.05).

### OANO_2_, a Pleiotropic-TRP Agonist, Has Mixed Inhibitory, and Facilitatory Effects on Guinea Pig SBCs

In contrast to its excitatory effect in rat bladder strips (Artim et al., 2011), OA-NO_2_ at 30 μM elicited either inhibitory or excitatory effects on SBCs (*n* = 48 strips from 5 male and 24 female guinea pigs). Inhibition was present in 77% of strips (Figure 1C), where it decreased the area under the curve (AUC) by 21 ± 3%, (*P* < 0.001) measured 0–5 min after application, while excitation occurred in 23% of strips (Figure 1F), where it significantly increased the AUC by 28 ± 9% (*P* < 0.01). Neither response was accompanied by a significant change in the baseline tension (*P* > 0.05; Figures 1C,F). The inhibitory and excitatory effects reached a maximum at 2–3 min, after application and the effects persisted for 25–30 min and 5 min, respectively (*P* < 0.05). Oleic acid (30 μM), a non-electrophilic fatty acid control, did not alter AUC or baseline tension (*P* > 0.05, *n* = 5 strips from three animals; data not shown).

### OAG, a TRPC Agonist, Reduces SBCs

Oleyl-acetyl-glycerol (OAG, 50 μM), a TRPC3,6,7 channel agonist (Venkatachalam et al., 2003) gradually suppressed SBCs, eliciting a small (11%) but not significant reduction at 10-15 min and a maximal reduction (29 ± 5%) at 40–45 min after application (*P* < 0.001, *n* = 10–12 strips from 1 male and 6 female guinea pigs, Figure 1D). Baseline tension was significantly decreased by 23 ± 8% at the time of maximum inhibition (40–45 min, *P* < 0.001; Figure 1D).

### Effect of Tetrodotoxin (TTX, 1 μM) on Agonist Evoked Responses

**Figure 2:**
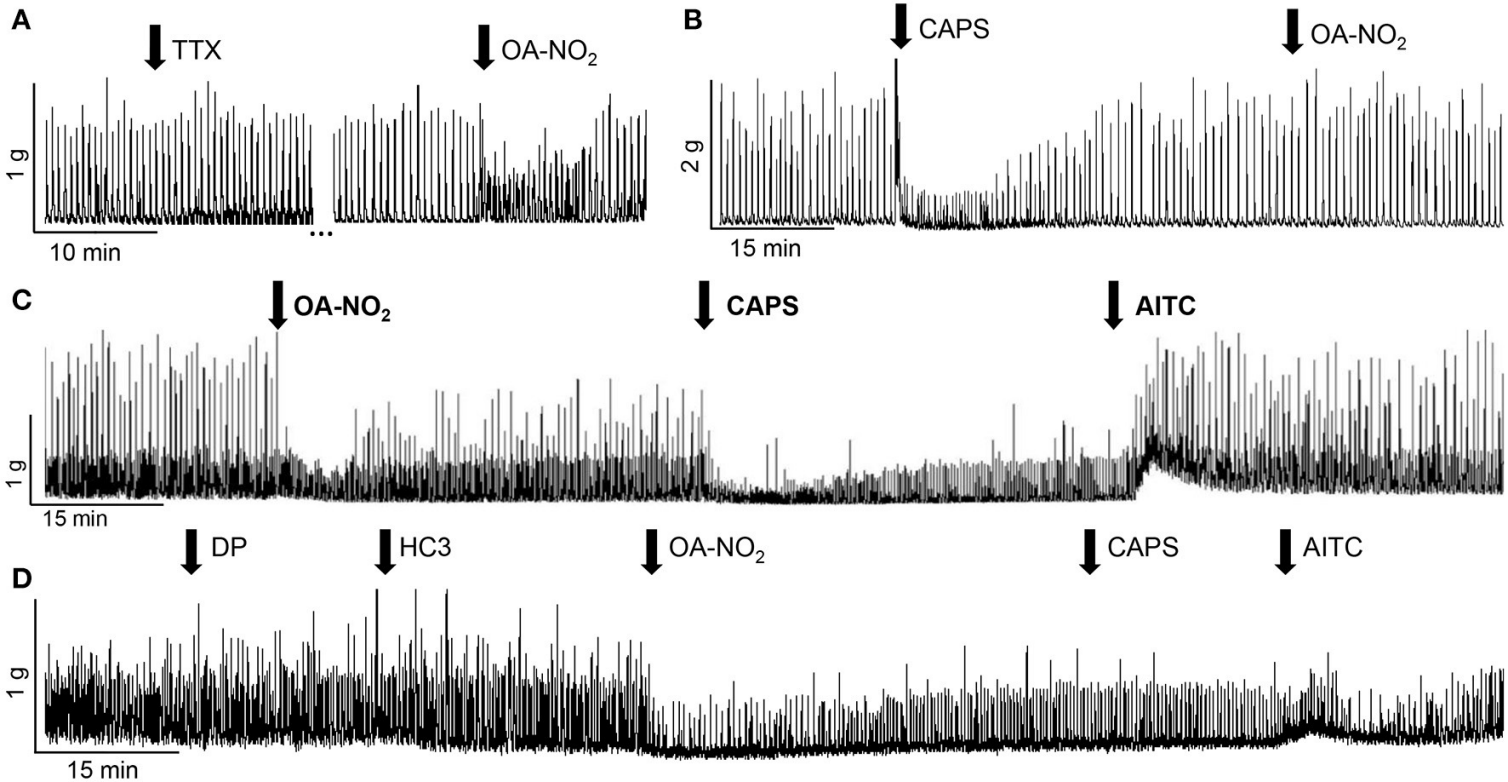
Effects of various agents on SBCs in guinea pig bladder strips. **(A)** Tetrodotoxin (TTX, 1 μM) does not alter SBCs or the inhibitory effect of OA-NO_2_ (30μM). The gap (…) in recording represents 24 min. **(B)** Pretreatment with CAPS (1 μM) blocks the inhibition elicited by the subsequent administration of OA-NO_2_ (30 μM) in the same bladder strip. **(C)** Inhibitory responses of OA-NO_2_ (30 μM) and CAPS (1 μM), and the excitatory response of AITC (100 μM) elicited in the same bladder strip by sequential application of the three agents, applied at the arrows and maintained in the tissue bath. **(D)** A combination of antagonists for TRPV1 (diarylpiperazine, DP, 1 μM) and TRPA1 (HC3-030031, HC3, 10 μM) does not alter the inhibitory effect of OA-NO_2_ (30 μM) but suppresses the effects on SBCs of the subsequent administration of CAPS (1 μM) and AITC (100 μM) in the same bladder strip. Calibration bars refer to time (min) and tension (gram, g).

**Figure 3:**
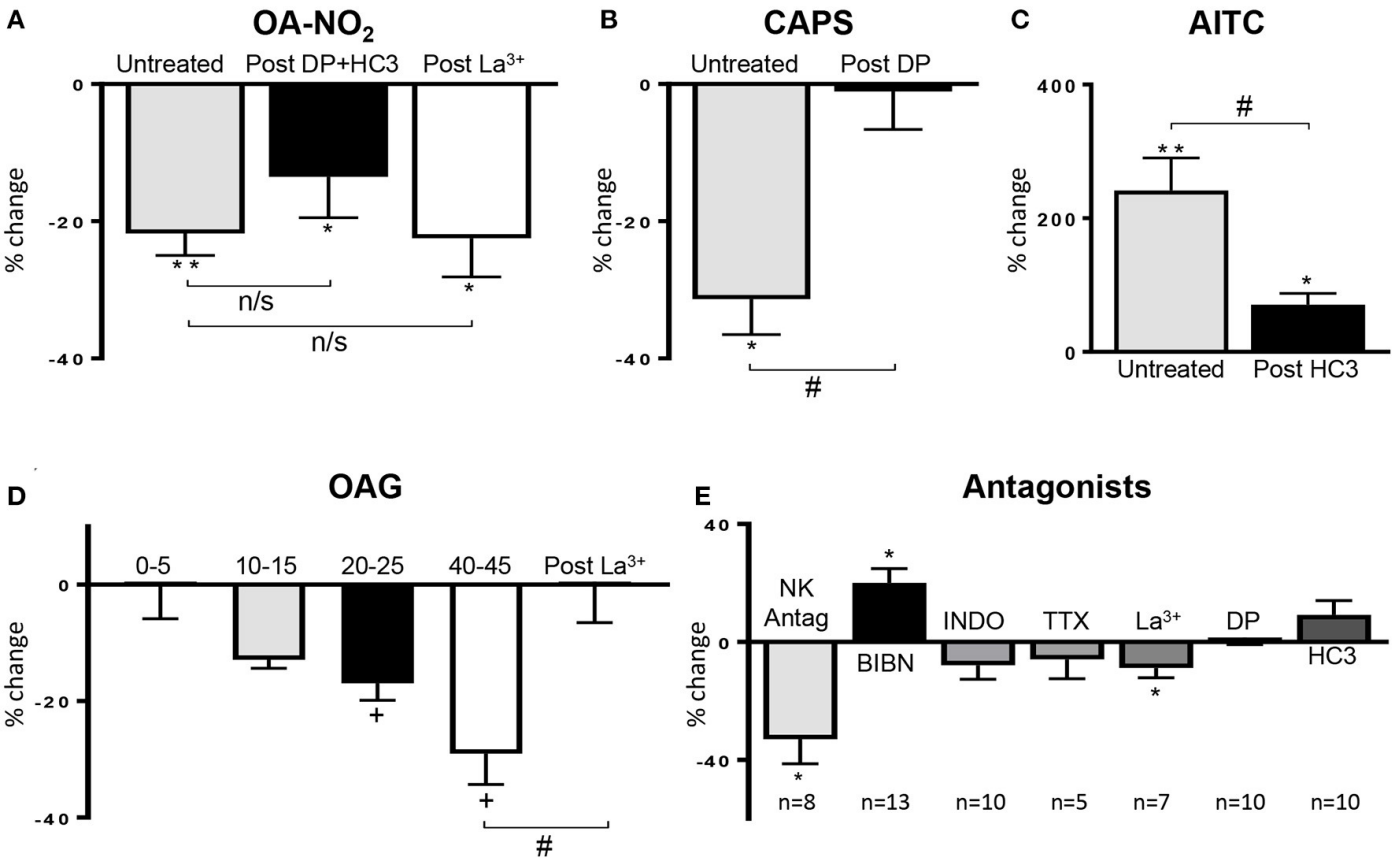
**(A)** Summary data showing that the OA-NO_2_ inhibitory response is not blocked by a combination of a TRPV1 (diarylpiperazine, DP) and a TRPA1 antagonist (HC3; *n* = 16) or the TRPC antagonist (La^3+^, 50 μM; *n* = 9). **(B,C)** Summary data showing that DP and HC3 suppress the effects of CAPS (*n* = 12) and AITC (*n* = 16), respectively. **(D)** Summary data showing the time course of the inhibitory effect of OAG (*n* = 10–12) and the block of this effect by La^3+^ (50 μM; *n* = 6), 45 min after application of OAG. **(E)** Effect of various antagonists on SBC AUC shown as % change from control SBC activity before treatment. Neurokinin receptor antagonists (NK Antag), a combination of three antagonists subtypes (1, 2 and 3: SR 140333, SR 48968 and SR 142801, respectively, 1 μM of each drug); BIBN (25 μM, CGRP receptor antagonist), indomethacin (INDO, 500 nM, COX inhibitor), concentrations of TTX, La^3+^, DP, and HC3 are the same as those indicated in Figure 2. Data was obtained from single experiments with either a pristine agonist or an antagonist followed by an agonist and graphs are presented as % change in SBC area under the curve (AUC) compared to basal activity. Statistics were performed on raw numbers: **(A–C)** 2-tailed *t*-test, *P < 0.05 and **P < 0.001, and a two-way ANOVA with Bonferroni multiple comparisons *post-hoc* tests (brackets), ^#^*P* < 0.05; **(D)** one-way ANOVA with Dunnett's multiple comparison *post-hoc* test (OAG; ^+^*P* < 0.05) and two-way ANOVA (bracket) with Bonferroni multiple comparisons *post-hoc* test (La^3+^; ^#^*P* < 0.05); **(E)** 2-tailed *t*-test, *P < 0.05, compared with basal activity; *n*, number of strips].

**Figure 4:**
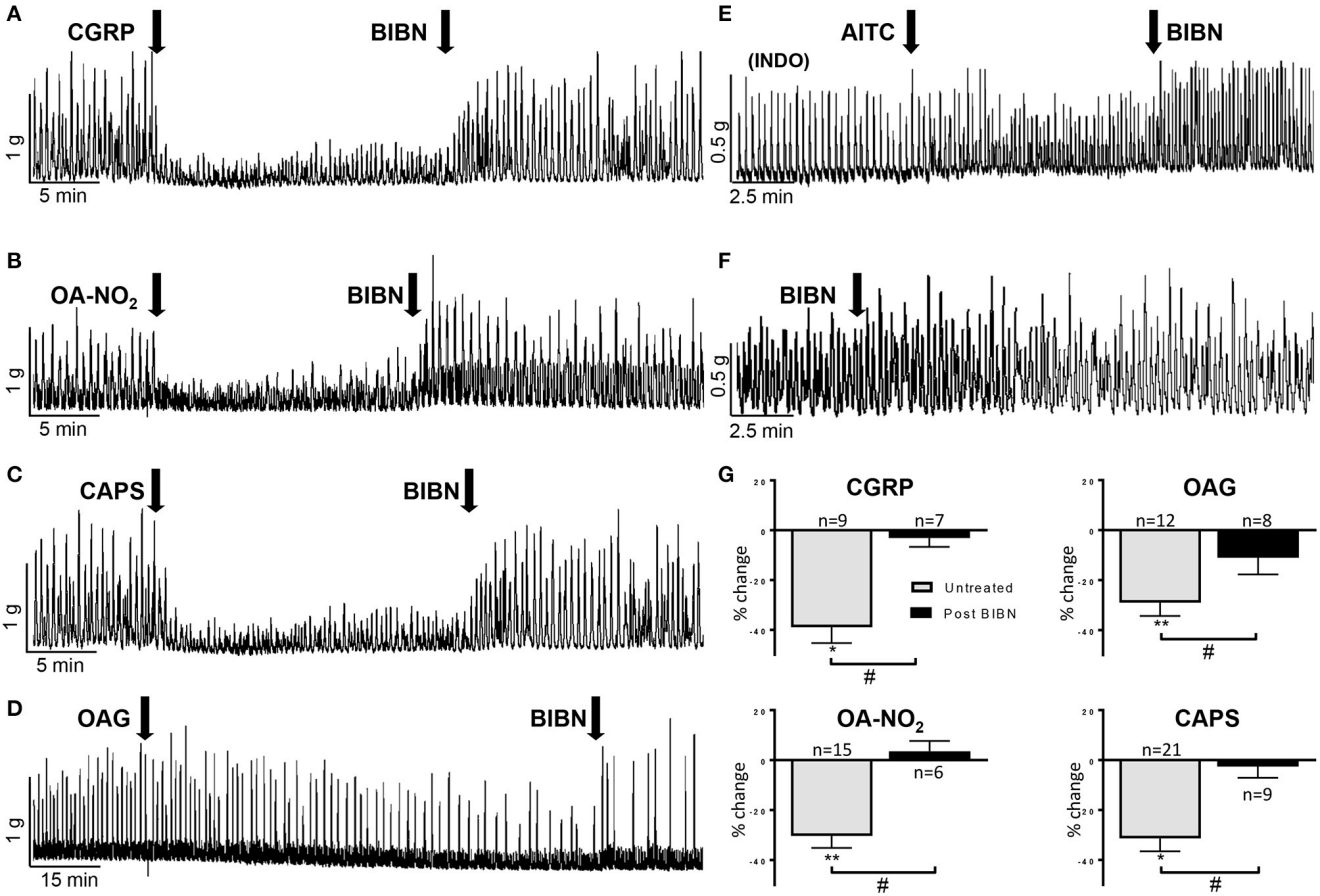
**(A–D)** BIBN (25 μM) a CGRP antagonist reverses the inhibition of SBCs elicited by CGRP (100 nM), OA-NO_2_ (30 μM), CAPS (1 μM), and OAG (50 μM). **(E)** Pretreatment with indomethacin (INDO, 500 nM) average of 25 min before application of AITC blocks the excitatory effect of AITC (100 μM) on SBCs and unmasks an inhibitory effect that is reversed by BIBN (25 μM; see Figure 5H for graph). **(F)** BIBN (25 μM) alone elicits a gradual increase in the amplitude of SBCs. **(G)** Graphs summarizing the magnitude of the inhibition of SBCs (% change of AUC) elicited by CGRP, OAG, OA-NO_2_, and CAPS and the block of the inhibition by pretreatment of BIBN. Statistics were performed on raw numbers: 2-tailed *t*-test, *P < 0.05 and **P < 0.001 and a two-way ANOVA with Bonferroni multiple comparisons *post-hoc* test (brackets), ^#^*P* < 0.05; *n*, number of strips.

To examine the role of efferent neurons or an intramural neural network, in the responses of TRP channel agonists the agonists were administered in the presence of TTX. TTX did not significantly alter: [1] control SBCs (five strips from two guinea pigs; Figures 2A, 3E), [2] the excitatory effect of AITC (100 μM, three strips from three guinea pigs, 124 ± 30% increase in AUC, data not shown), [3] the inhibitory effect of OA-NO_2_ (three strips from three guinea pigs, 15 ± 11% decrease of AUC, Figure 2A) or [4] the inhibitory effect of CAPS (1 μM, six strips from three guinea pigs, 19 ± 7% decrease in AUC, data not shown). However, the initial transient excitatory effect of CAPS which preceded the inhibition was blocked by TTX (*P* < 0.05, *n* = 6 strips from three guinea pigs, 19.5 ± 18% decrease of AUC, measured 30 s after application of CAPS, data not shown).

### Desensitization of Agonist Responses

In a separate group of experiments, agonists were applied multiple times to the same bladder strip after washout and long recovery periods (60–100 min). Neither OA-NO_2_ or CAPS elicited responses during the second application (*n* = 5 and 9 strips from three and four guinea pigs respectively, *P* < 0.05, data not shown). On the other hand, the second application of AITC elicited a response (203 ± 62% increase in the SBCs AUC, *n* = 5 strips), that was not significantly different (*P* > 0.05) from that elicited by the first application (data not shown).

In another group of experiments where multiple agents were administered sequentially to the same bladder strip (Figure 2) application of CAPS blocked the responses to the subsequent application of OA-NO_2_, even at a time point when the SBCs had recovered from the initial inhibitory effect of CAPS (Figure 2B, *n* = 14 strips from 11 guinea pigs, *P* < 0.01). On the other hand, OA-NO_2_ did not alter the inhibitory response to the subsequent application of CAPS (Figure 2C, *n* = 17 strips from 13 guinea pigs). Administration of AITC during the inhibition induced by the combined application of OA-NO_2_ and CAPS rapidly reversed the inhibition (43 ± 15% increase in SBCs AUC, Figure 2C, *n* = 13 strips from seven guinea pigs).

### Effects of TRPA1, TRPV1, and TRPC Antagonists

After application of a combination of diarylpiperazine (DP, 1 μM), a TRPV1 antagonist and HC3-03001 (HC3, 10 μM), a selective TRPA1 receptor antagonist, at concentrations used in previous studies (Sculptoreanu et al., 2010; Artim et al., 2011; Zhang et al., 2014a) the bimodal facilitatory-inhibitory effect of CAPS was eliminated (Figure 3B, *n* = 12 strips from five guinea pigs) and the facilitatory response of AITC was significantly reduced (Figure 3C, *n* = 16 strips from four guinea pigs). Application of DP or HC3 alone or in combination did not change the SBCs (*n* = 10 strips from four guinea pigs; Figures 2D, 3E).

The inhibitory effect of OA-NO_2_ was not blocked by TRPV1 (DP, 1 μM) or TRPA1 (HC3, 10 μM) antagonists (18 ± 6% decrease, *n* = 16 strips from five guinea pig; Figures 2D, 3A). In the presence of a low concentration of lanthanum chloride (La^3+^, 50 μM), an agent known to block various subtypes of TRPC channels (Clapham et al., 2005) OA-NO_2_ still decreased AUC by 21 ± 5% (Figure 3A, *n* = 9 strips from six guinea pigs), whereas, 50 μM La^3+^ blocked OAG suppression of SBC AUC (*n* = 6 strips from five guinea pigs; Figure 3D). La^3+^ (50 μM) alone slightly decreased AUC (Figure 3E; 9 ± 3%, *n* = 7 strips from four guinea pigs; *P* < 0.05) but did not affect baseline tension (−1 ± 1%, *P* > 0.05). Higher concentrations of La^3+^ could not be tested because they further suppressed SBCs.

### Release of CGRP Mediates OA-NO_2_, OAG, and Capsaicin (CAPS) Induced Inhibition

CGRP released from bladder C-fiber afferents has been reported to suppress spontaneous contractions of the guinea pig bladder (Maggi, 1993; Gillespie et al., 2006; Lee et al., 2016). In our experiments, CGRP (100 nM) significantly reduced the AUC and baseline tension by 39 ± 6% and 11 ± 6% respectively (*n* = 9 strips from nine guinea pigs, *P* < 0.001) and this inhibition was reversed (Figure 4A) or blocked in the presence of a CGRP antagonist (BIBN, 25 μM; *n* = 7 strips from three guinea pigs, 3 ± 6% decrease; Figure 4G) (Doods et al., 2000). To test the possibility that the inhibitory effects of other agonists on SBCs were mediated by release of CGRP, bladder strips were treated with BIBN (25 μM) before (Figure 4G) and after OA-NO_2_, OAG, or CAPS application (Figures 4B–D). BIBN blocked the inhibitory effects of OA-NO_2_ (*n* = 6 strips from four guinea pigs, 4 ± 4%), CAPS (*n* = 9 strips from five guinea pigs, 3 ± 5%) and OAG (*n* = 8 strips from four guinea pigs, 11 ± 7% decrease; Figure 4G). However, the transient facilitatory effect of CAPS which was detected in the majority of untreated preparations was similar in magnitude (172% increase in SBCs AUC) and duration in preparations treated with BIBN (*n* = 9 strips, data not shown). Thus, the block of inhibition by BIBN did not unmask prolonged facilitatory effects of CAPS or OANO_2_ that occurred in the minority of untreated preparations (Figures 1E,F). BIBN (25 μM) administered alone increased SBC AUC by 20 ± 5% (*P* < 0.001, *n* = 13 strips from five guinea pigs, Figures 3E, 4F) suggesting that in the distended bladder there is tonic release of CGRP which inhibits bladder contractility and that OA-NO_2_, CAPS, and OAG inhibit SBCs by increasing the release of CGRP.

### Release of Substance P Is Not the Primary Mechanism of AITC Induced Excitation

**Figure 5:**
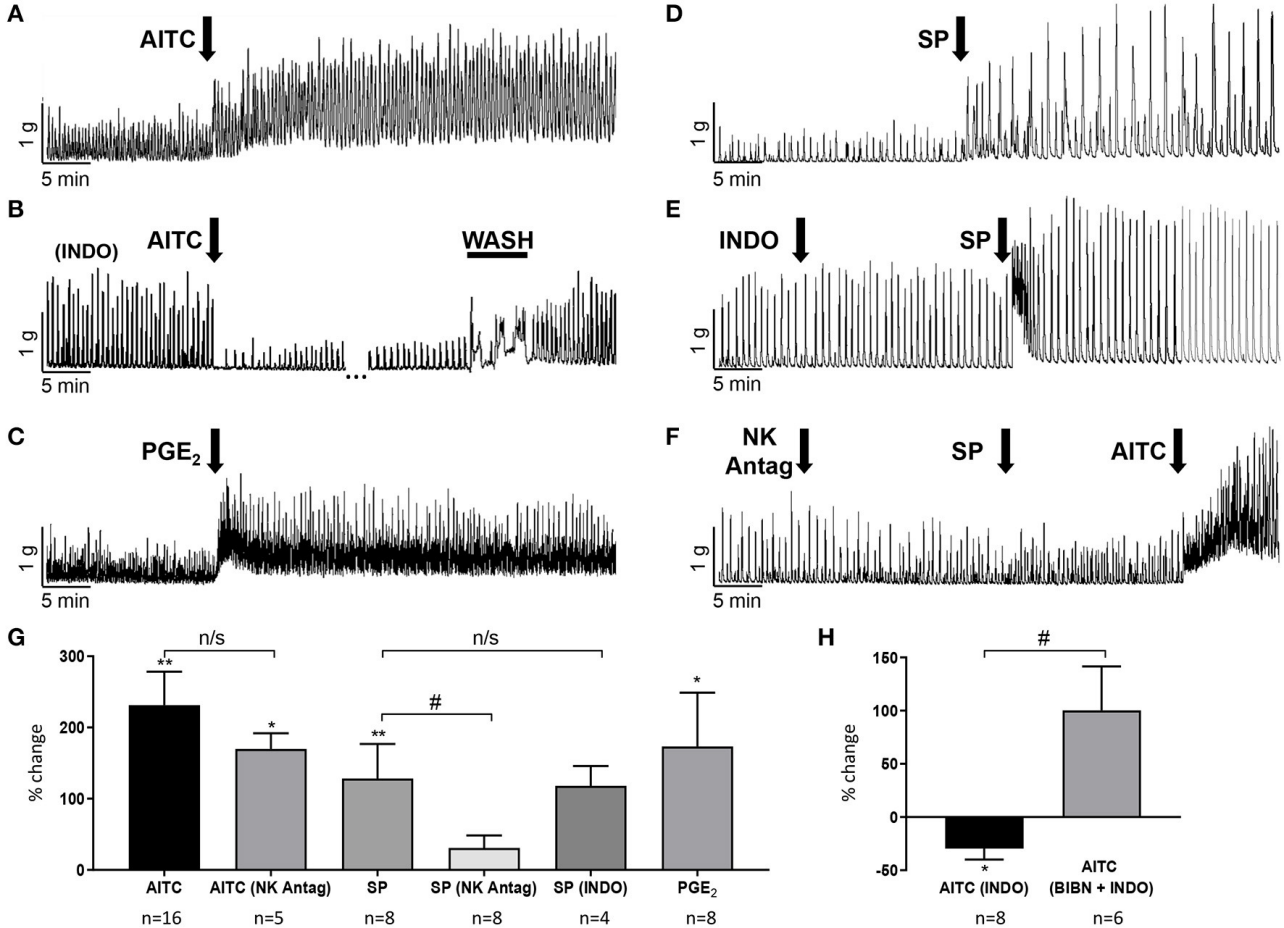
AITC (100 μM) enhancement of SBCs is mediated by the release of prostaglandins (PG) and not by the release of substance P (SP). **(A)** AITC enhances SBCs and this effect is reversed to inhibition by pretreatment with indomethacin (INDO, 500 nM) **(B)** which is in turn reversed by a wash (gap in record represents 24 min). **(C)** Prostaglandin E2 (PGE_2_, 500 nM) mimicks the excitatory effect of AITC. **(D)** SP (1 μM) enhances SBCs; however, this effect is not blocked by INDO **(E)** but is blocked by a cocktail of neurokinin receptor 1, 2, and 3 antagonists (1 μM each) which does not block the excitatory effect of AITC **(F)**. **(G)** Effects of agonists and antagonists shown in **(A–F)** are summarized as % change in SBC AUC compared to basal activity, data were obtained from single experiments with either a pristine agonist or an antagonist followed by an agonist **(H)**. After indomethacin (INDO) treatment AITC reduces SBC and this effect is reversed by BIBN as shown in Figure 4E. Statistics were performed on raw numbers: **(G,H)**, 2-tailed *t*-test, *P < 0.05 and **P < 0.001, and a two-way ANOVA with Bonferroni multiple comparisons *post-hoc* tests were performed to compare the antagonist effects (brackets), ^#^*P* < 0.05; *n*, number of strips.

Substance P (SP) released from bladder afferents contributes to the excitatory effects of CAPS, OA-NO_2_, and AITC on SBC in rat bladder strips (Andrade et al., 2006; Artim et al., 2011). In the present experiments, application of SP (1 μM) mimicked the excitatory effect of AITC on spontaneous contractile activity (Figures 5A,D) significantly increasing SBC AUC by 128 ± 48% (*P* < 0.01) but only slightly increased baseline tension (5 ± 2%, *P* < 0.05; Figure 5D, *n* = 8 strips from five guinea pigs). To determine the role of SP release in AITC induced excitation, bladder strips were pretreated with a combination of neurokinin receptor 1, 2, and 3 antagonists (SR 140333, SR 48968, and SR 142801; 1 μM each) which blocks the excitatory effect of substance P in the guinea pig ileum (Holzer et al., 1998; Takahashi et al., 2002). The neurokinin receptor antagonists alone (*n* = 8 strips from eight guinea pigs) decreased AUC by 33 ± 9% (*P* < 0.05), without changing the baseline tension and significantly reduced the SP induced enhancement of SBCs (*n* = 8 strips from seven guinea pigs, Figures 3E, 5F,G). However, pretreatment with the neurokinin receptor antagonists did not significantly change the AITC induced enhancement of SBCs (*n* = 5 strips from five guinea pigs, Figures 5F,G, *p* > 0.05) indicating that SP released from bladder afferents is not the primary mechanism for AITC induced excitatory responses.

### Release of Prostaglandin E_2_ Contributes to AITC Induced Excitation

Consistent with the results from other studies in guinea pig bladder (Nile et al., 2010; Rahnama'i et al., 2013; Parajuli et al., 2014), application of prostaglandin E_2_ (500 nM) significantly increased the SBC AUC by 173 ± 76% as well as the baseline tension by 66 ± 40% (*P* < 0.05, *n* = 8 strips from six guinea pigs; Figures 5C,G). To determine if prostaglandins contribute to the excitatory effects of AITC, indomethacin (INDO, 500 nM), a non-selective cyclooxygenase (COX) inhibitor that suppresses prostaglandin synthesis was applied prior to AITC treatment. INDO alone did not significantly alter the SBC AUC (8 ± 5% decrease) or baseline tension (4 ± 1% decrease, *n* = 10 strips from eight guinea pigs, measured at 10–15 min after application; Figures 3E, 5E), however, pretreatment with INDO reversed the AITC induced excitatory response to inhibition (Figure 5B, a 27 ± 9% reduction in SBC AUC with no change in baseline tension, 0.2 ± 3%, *n* = 8 strips from six guinea pigs, *P* < 0.05). BIBN (25 μM) blocked the AITC inhibition unmasked by INDO or converted the inhibition to excitation (100 ± 42% increase in AUC and 11 ± 7% increase in baseline tension; *n* = 6 strips from five guinea pigs; Figures 4E, 5H). INDO did not alter the SP induced excitation (118 ± 28% increase in AUC; *n* = 4 strips from four guinea pigs; Figures 5E,G).

## Discussion

This study revealed that activation of TRP channels in guinea pig urinary bladder can either enhance or suppress spontaneous contractile activity by releasing prostaglandins or CGRP, respectively. The main findings are: [1] a TRPA1 agonist (AITC) enhances SBCs by releasing prostaglandins. This effect is blocked by indomethacin (INDO) a COX inhibitor. AITC also inhibits SBCs by releasing CGRP an effect blocked by BIBN, a CGRP receptor antagonist. [2] A TRPV1 agonist (CAPS), and TRPC agonists (OA-NO_2_ or OAG) suppress the contractile activity in the majority of bladder strips by releasing CGRP [3]. The inhibitory effect of CAPS is preceded by a transient facilitation that is blocked by TTX and therefore likely mediated by activation of neurons in the bladder wall.

The mechanisms underlying the effects of the TRP agonists on bladder activity are commonly ascribed to activation of transmitter release from intramural afferent nerves because: [1] TRP channels are expressed in bladder afferent neurons (Maggi, 1993; Franken et al., 2014; Shimizu et al., 2017, 2018), [2] the TRP agonists are known to activate primary afferent neurons in the guinea pig (Zhang et al., 2014a), [3] CGRP, a peptide neurotransmitter synthesized and released by a large percentage of visceral afferent nerves (Maggi et al., 1988b; Keast and de Groat, 1992; Hwang et al., 2005; Shimizu et al., 2017), is involved in the CAPS (Maggi et al., 1988b) and OA-NO_2_ induced inhibition of bladder activity (Figures 1A,B), [4] CGRP release in the guinea pig bladder by CAPS or bradykinin is reduced by desensitization of afferent nerves following local or systemic administration of CAPS (Maggi et al., 1988a, 1989), [5] neuropeptides released from bladder afferent nerves modulate smooth muscle activity (Maggi and Meli, 1986; Artim et al., 2011; Franken et al., 2014), and [6] synthesis of prostaglandin can be increased in the urinary bladder by distension or noxious stimuli that activate afferent nerves (Bjorling et al., 1994). However, TRP channels are also expressed in other cells in the bladder, e.g., smooth muscle cells and cells in the urothelial and lamina propria layers of the mucosa (Birder et al., 2001; Kullmann et al., 2009; Everaerts et al., 2010; Yu et al., 2011), which can also release neurotransmitters that influence smooth muscle activity. Because prostaglandins enhance tonic and phasic contractions of isolated mucosal tissue (Stromberga et al., 2020), it is possible that AITC release of prostaglandins in full thickness bladder strips facilitates smooth muscle activity in part via an indirect route involving the mucosa. Thus additional experiments showing: [1] that effects of the agonists are abolished by elimination of the afferent innervation of the bladder or [2] that the responses persist in bladder strips after removal of the mucosa are needed to establish that the agonists act solely via a neurogenic mechanism or if they act on multiple targets in the bladder.

**Figure 6:**
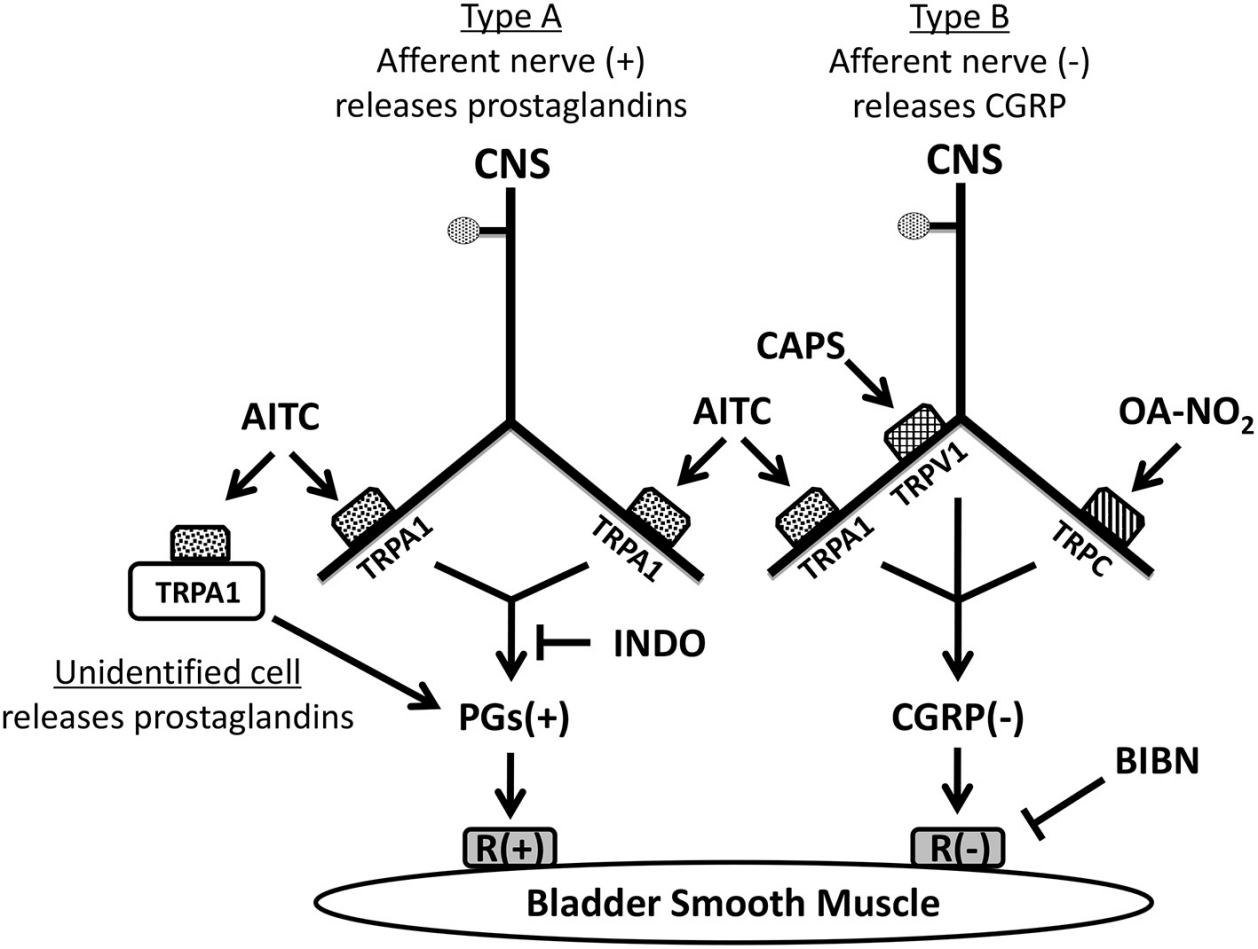
Pharmacological experiments suggest that at least two types of sensory nerves expressing TRP channels innervate guinea pig urinary bladder. TRPA1 channels are located on one population of nerves (Type A). Activation of TRPA1 in these nerves and/or unidentified non-neuronal cells by AITC excites bladder smooth muscle; an effect blocked by indomethacin indicating that the excitation is mediated by the synthesis and release of prostaglandins (PGs). Another population of sensory nerves (Type B) expresses TRPV1, TRPA1, and TRPC channels. Activation of these channels by capsaicin (CAPS), AITC, or OA-NO_2_ releases CGRP causing inhibition of the bladder smooth muscle. However, in comparison to the prominent effects of CAPS and OA-NO_2_ the inhibitory effect of AITC mediated by CGRP release from Type B nerves is relatively weak and only detectable when prostaglandin (PG) synthesis is blocked by indomethacin indicating that it is normally masked by the PG excitation. (+): excitatory; (–): inhibitory.

Assuming that afferent nerves are the principal target for the agonists (Figure 6), it is likely that TRPV1, TRPA1, and TRPC channels are co-expressed by one population of afferent nerves that produces bladder inhibition by releasing CGRP which is known to suppress spontaneous contractions of the guinea pig bladder (Maggi et al., 1988a; Giuliani et al., 1992; Gillespie, 2005; Lee et al., 2016). CAPS releases CGRP in the guinea pig bladder by a calcium dependent mechanism (Maggi, 1991, 1993) and calcium imaging experiments revealed that a percentage of OA-NO_2_ responsive guinea pig DRG neurons also respond to CAPS indicating co-expression of TRPV1 and TRPC channels in these neurons (Zhang et al., 2014a). The present experiments showed that pretreatment with CAPS suppresses the inhibitory effect of a subsequent administration of OA-NO_2_ (Figure 2B) further suggesting the co-expression of TRPV1 and TRPC.

CGRP could act at multiple sites to modulate the contractile activity of the bladder strips. CGRP has excitatory or facilitatory effects on peripheral (Cornelissen et al., 2001) and central neurons (Fisher et al., 1983; Han et al., 2010) and therefore it could excite intrinsic neural or neural-interstitial cell networks in the guinea pig bladder (Gillespie et al., 2006) to release inhibitory transmitters. However, a neural mechanism seems unlikely because tetrodotoxin (TTX), which blocks nerve activity, did not alter the spontaneous activity of the bladder strips or the inhibitory responses to OA-NO_2_ or CAPS (Figures 2A, 3E). Thus, CGRP released by TRP agonists is likely to act directly on bladder smooth muscle by stimulating the synthesis of cyclic AMP (Maggi et al., 1988b; Russell et al., 2014).

AITC has facilitatory and inhibitory effects on bladder activity (Figure 5). The facilitatory effect (Figure 1A) was blocked by pretreatment with the COX inhibitor, INDO (Figures 4E, 5H) and reduced by a TRPA1 receptor antagonist (HC3; Figures 2D, 3C) indicating that TRPA1 channels are expressed on a second population of afferents and/or on unidentified non-neuronal cells that release prostaglandins (Figure 6) that in turn excite the bladder smooth muscle (Figure 5C). However, in contrast to the action of AITC in the rat bladder where the excitatory effects are suppressed by neurokinin receptor antagonists (Andrade et al., 2006; Artim et al., 2011) the effects in the guinea pig bladder were unaffected by a combination of antagonists targeting neurokinin receptor subtypes 1, 2, and 3. In the presence of INDO, AITC decreased bladder contractions, an effect blocked by BIBN (Figures 4E, 5H) indicating that AITC activates TRPA1 receptors that may be co-localized with TRPV1 and TRPC receptors on afferent nerves releasing CGRP (Figure 6). This inhibitory effect of AITC mediated via CGRP release was clearly masked by the more prominent excitatory effect mediated by prostaglandins.

The distribution of TRPA1 channels in two populations of bladder afferents (Figure 6) is consistent with electrophysiological studies of afferents in the guinea pig bladder (Zagorodnyuk et al., 2007, 2009; Nicholas et al., 2017) which revealed five populations of muscle and mucosal mechanosensitive afferents based on the threshold to stretch and the sensitivity to AITC and CAPS. In these studies of urothelium free bladder preparations, low threshold stretch-sensitive afferents respond to AITC but only a small proportion of these afferents respond to CAPS (Nicholas et al., 2017), corresponding to the type A afferent population we show in Figure 6. However, a large percentage (72–86%) of high threshold stretch-sensitive afferents respond to AITC or CAPS, corresponding to the type B afferent population we show in Figure 6.

In the majority of strips CAPS produced a small initial transient enhancement followed by the CGRP mediated inhibition. A similar bimodal effect of CAPS in guinea pig bladder preparations was reported in earlier studies (Maggi et al., 1987; Gillespie, 2005). The transient facilitation but not the inhibition was blocked by pretreatment with TTX suggesting that the facilitatory effect of CAPS is mediated by activation of efferent neurons or an intramural neural network that is known to be present in the guinea pig bladder (Gillespie et al., 2006; Eastham and Gillespie, 2013). The transmitters involved in this facilitatory response were not examined. The TTX resistant release of CGRP is presumably elicited by the ligand gated increase in intracellular Ca^2+^ in the sensory nerve terminals or non-neural cells.

TRPV1 and TRPA1 antagonists did not change spontaneous activity (Figures 2D, 3E); however, the CGRP antagonist, BIBN, significantly increased the contractions (Figures 3E, 4F) indicating the existence of tonic CGRP inhibition. This inhibition might be induced by CGRP released from mechanosensitive afferent nerves in response to stretch of the bladder strips and/or the ongoing intrinsic contractions. Spontaneous contractile activity was also significantly reduced by application of a combination of three neurokinin receptor subtype antagonists (Figures 3E, 5F) indicating that neurokinins released from afferent nerves generate a tonic facilitation of spontaneous contractile activity. The tonic release of neurokinins may occur in a third type of afferent other than the two shown in Figure 6.

### Limitations of This Study

The main limitation of our study is the use of a single technique, i.e., the measurement of spontaneous contractions of bladder strips and the use of only pharmacological approaches to examine the functions of TRP channels in the bladder. More detailed information about the distribution of TRP channels in the bladder will be obtained in future experiments using immunohistochemical techniques. Mucosa free bladder strips will also be used to limit the possible sites of drug action.

### Perspectives and Significance

In summary, activation of TRP channels in guinea pig bladder strips enhances or suppresses SBCs. It is likely that one mechanism underlying these effects is release of signaling molecules from bladder afferent nerves which express multiple TRP channels. Activation of TRPV1, TRPA1 and TRPC channels expressed by one type of afferent nerve releases CGRP that elicits inhibition; while activation of TRPA1 channels in another type of afferent nerve and/or in non-neuronal cells releases prostaglandins that elicit excitation (Figure 6). CGRP and neurokinins which are tonically released are involved in an ongoing inhibition and facilitation, respectively, of SBCs that occurs in the absence of TRP stimulation. This suggests that a third type of mechanosensitive afferent nerve releases neurokinins in response to stretch. These findings further indicate that bladder afferent nerves have complex inhibitory and facilitatory efferent functions in addition to their primary role in bladder sensation and that they release multiple transmitters that modulate bladder smooth activity to influence the storage and elimination of urine. The type B nociceptive afferent population is unusual because it presumably co-releases CGRP with glutamate as excitatory transmitters in the spinal cord (Bird et al., 2006) that can potentially activate central micturition pathways and induce bladder overactivity; while simultaneously releasing CGRP which acts as an inhibitory transmitter in the periphery to suppress spontaneous bladder contractions. It is not known if these two functions can be turned on independently. The ability of OANO_2_, to selectively target the type B afferent population and induce CGRP mediated inhibition combined with its known anti-inflammatory actions might generate a synergistic interaction that could result in an effective treatment for some types of overactive bladder dysfunction.

## Data Availability Statement

The raw data supporting the conclusions of this article will be made available by the authors, without undue reservation.

## Ethics Statement

The animal study was reviewed and approved by Ethics Committee of Pittsburg University.

## Author Contributions

WG, BF, and XZ designed experiments. SD, KK, and JL carried out experiments and analyzed data. JB, SW, and XZ wrote the manuscript. WG critically edited the manuscript. All authors contributed to the article and approved the submitted version.

## Conflict of Interest

The authors declare that the research was conducted in the absence of any commercial or financial relationships that could be construed as a potential conflict of interest.

## Abbreviations

AITC: allyl isothiocyanate
SBCs: spontaneous bladder contractions
AUC: area under the curve
BLT: baseline tension
BIBN: olcegepant
CAPS: capsaicin
CGRP: calcitonin gene-related peptide
DP: diarylpiperazine
DRG: dorsal root ganglia
HC3: HC3-03001
INDO: indomethacin
La^3+^: lanthanum
OAG: oleoyl-2-acetyl-sn-glycerol
OA-NO_2_, 9- and 10-nitro-octadec-9-enoic acid: nitro-oleic acid
PG: prostaglandin
SP: substance P
TTX: tetrodotoxin.
